# Research on the integration development model of “language + culture + tourism + older adults care” based on digital technology

**DOI:** 10.3389/fpubh.2026.1775237

**Published:** 2026-04-10

**Authors:** Rui Wang, Zhaoxi Li

**Affiliations:** Chengde College of Applied Technology, Chengde, China

**Keywords:** cultural participation, digital integration, language accessibility, older adults services, smart aging

## Abstract

This study empirically examines the effectiveness of integrating “language + culture + tourism + older adults care” services through digital technology to address the challenges of population aging. Using public data from six Chinese cities (Shanghai, Hangzhou, Wuhan, Changsha, Chengdu, Xi’an) collected from June to August 2024, we constructed a Digital Integration Index (DII) and employed structural equation modeling and multiple regression analysis to evaluate integration effects. Results demonstrate that digital integration significantly improves older adults people’s quality of life (effect size = 0.68), digital participation (0.74), and service satisfaction (0.71), with synergistic effects contributing approximately one-third of total effects. The integration model shows stronger effects for the 60–65 age group (0.82) and eastern cities (0.79), though central and western regions exhibit greater improvement magnitude. Key success factors include improved dialect recognition accuracy (68 to 89%) and age-friendly interface design. Cost–benefit analysis reveals a 24.3% reduction in per-user service costs post-integration. The study provides quantitative evidence for multi-domain service integration, offering an evidence-based framework for policy-making. Future research should extend observation periods and expand geographic coverage to validate long-term effects and rural applicability.

## 1 Introduction

With the acceleration of global population aging, the quality of life and social participation of the older adults population have become focal points for governments and academia worldwide (1, 2). According to World Health Organization data, by 2050, the global population aged 60 and above will reach 2 billion, accounting for 22% of the total population (3, 4). The COVID-19 pandemic has further highlighted age-related vulnerabilities in health outcomes (5). Simultaneously, digital transformation is profoundly changing the delivery methods of social services and the lifestyle patterns of the older adults. However, older adults groups face multiple challenges in enjoying digital conveniences, among which the digital divide (6), language barriers (7), cultural adaptation difficulties (8), tourism participation obstacles, and the fragmentation of older adults care services have become key factors constraining improvements in their quality of life.

As the country with the world’s largest older adults population, China had over 280 million people aged 60 and above by the end of 2023, accounting for 20.1% of the total population (1). China’s aging presents characteristics of rapid speed, large scale, and “getting old before getting rich” (3). Meanwhile, China possesses rich cultural tourism resources and a rapidly developing digital economy, providing unique practical scenarios for exploring the integrated development model of “language + culture + tourism + older adults care.” However, in reality, these four domains often operate in silos, lacking effective integration mechanisms, resulting in low resource utilization efficiency and difficulty in meeting the diversified needs of the older adults. The digital divide issue is particularly prominent among older adults groups (6), with surveys showing that internet users aged 60 and above account for only 12.2% of total internet users, far below their population proportion. Problems such as language comprehension difficulties (7, 8), cultural cognitive differences, barriers to tourism information access, and insufficient accessibility of older adults care services are intertwined, forming complex social challenges.

Existing research has explored issues related to digital inclusion, cultural participation, tourism behavior, and older adults care services for the older adults from different perspectives, but lacks a systematic integration perspective. In the field of cultural participation, recent studies indicate that participating in cultural activities can significantly improve older adults people’s cognitive function and social connections (9). However, existing cultural services face problems such as information asymmetry and high participation thresholds, and the potential of digital technology in connecting older adults people with cultural resources has not been fully explored (10). Older adults tourism research mainly focuses on travel motivations, destination selection, and satisfaction evaluation (11). Recent studies have examined how virtual reality and digital technologies can shape tourist behavior and enhance tourism experiences (12, 13). However, these studies generally overlook the impact of language barriers on older adults tourism experiences and how to improve tourism service accessibility through digital technology.

Older adults care service research has made progress in exploring service models across different countries (14) and examining the impacts of technological innovations like robots in older adults care institutions (15). Recent research has also highlighted the importance of service integration to address fragmentation issues. However, there is still a gap in understanding how to achieve effective integration of multiple service domains through digital platforms. The development of quasi-experimental designs (16) and implementation research methods (17) provides methodological foundations for evaluating such integration models.

Despite these advances, a critical research gap remains. Existing studies have examined digital inclusion, cultural participation, tourism behavior, and older adult care services largely in isolation, without systematically investigating how these domains interact when integrated through digital platforms. While the potential benefits of cross-domain integration have been theoretically proposed, empirical evidence quantifying the synergistic effects—and the mechanisms through which they operate—is notably absent. This gap is particularly significant given the substantial policy investments being made in “smart aging” initiatives across China and other rapidly aging societies, often without rigorous evidence regarding the added value of integration over standalone services. The present study addresses this gap by providing the first empirical test of a four-domain integration model, quantifying both direct and indirect effects, and identifying the conditions under which integration yields the greatest benefits.

To address these research gaps, a coherent theoretical foundation is essential for justifying the integration of these four specific domains. This study’s framework is grounded in the World Health Organization’s Active Ageing Framework, which identifies three interdependent pillars for quality of life in older age: health, participation, and security (18, 19). The health pillar directly corresponds to older adult care services, encompassing preventive health management and care coordination. The participation pillar—emphasizing continued engagement in social, cultural, and recreational activities—is operationalized through cultural participation and tourism participation, both of which maintain cognitive vitality and combat social isolation. Language accessibility serves as a foundational enabler cutting across all pillars; without adequate linguistic support, older adults facing dialect barriers or literacy challenges cannot effectively access health information, participate in cultural activities, or navigate tourism services (20).

The rationale for integration rather than isolated service delivery draws on Integrated Care Theory, which posits that fragmented services create access barriers and fail to address multidimensional needs (21, 22). Digital technology serves as the integrating mechanism through three pathways theorized in platform ecosystem literature (23, 24): data interoperability enabling seamless information sharing across domains, interface unification reducing cognitive load through a single age-friendly access point, and network effects creating value amplification as user engagement in one domain enhances service quality across all domains. This theoretical synthesis provides the conceptual foundation for our empirical investigation.

Based on the above background and research gaps, this study aims to empirically test the effectiveness of integrating the four domains of “language + culture + tourism + older adults care” based on digital technology. By constructing a measurable integration index system using advanced data analysis platforms, we verify the mechanism of digital platforms in promoting the coordinated development of the four domains and evaluate the impact of the integration model on older adults people’s quality of life, digital participation, and service satisfaction. Through comparing data changes before and after integration, this study will provide quantitative evidence of integration effects, offering an evidence-based framework for related policy-making and service design. Based on empirical analysis results, this study expects to propose actionable implementation paths and policy recommendations to promote multi-domain integrated development, ultimately improving older adults people’s quality of life and social participation.

## 2 Materials and methods

### 2.1 Research design and conceptual framework

This study adopts an empirical research design based on public data analysis, evaluating the integration effects of the four domains of “language + culture + tourism + older adults care” supported by digital technology through integrating government open data, public statistical information, and network-accessible data. The research timeframe spans from March to December 2024.

**Figure 1:**
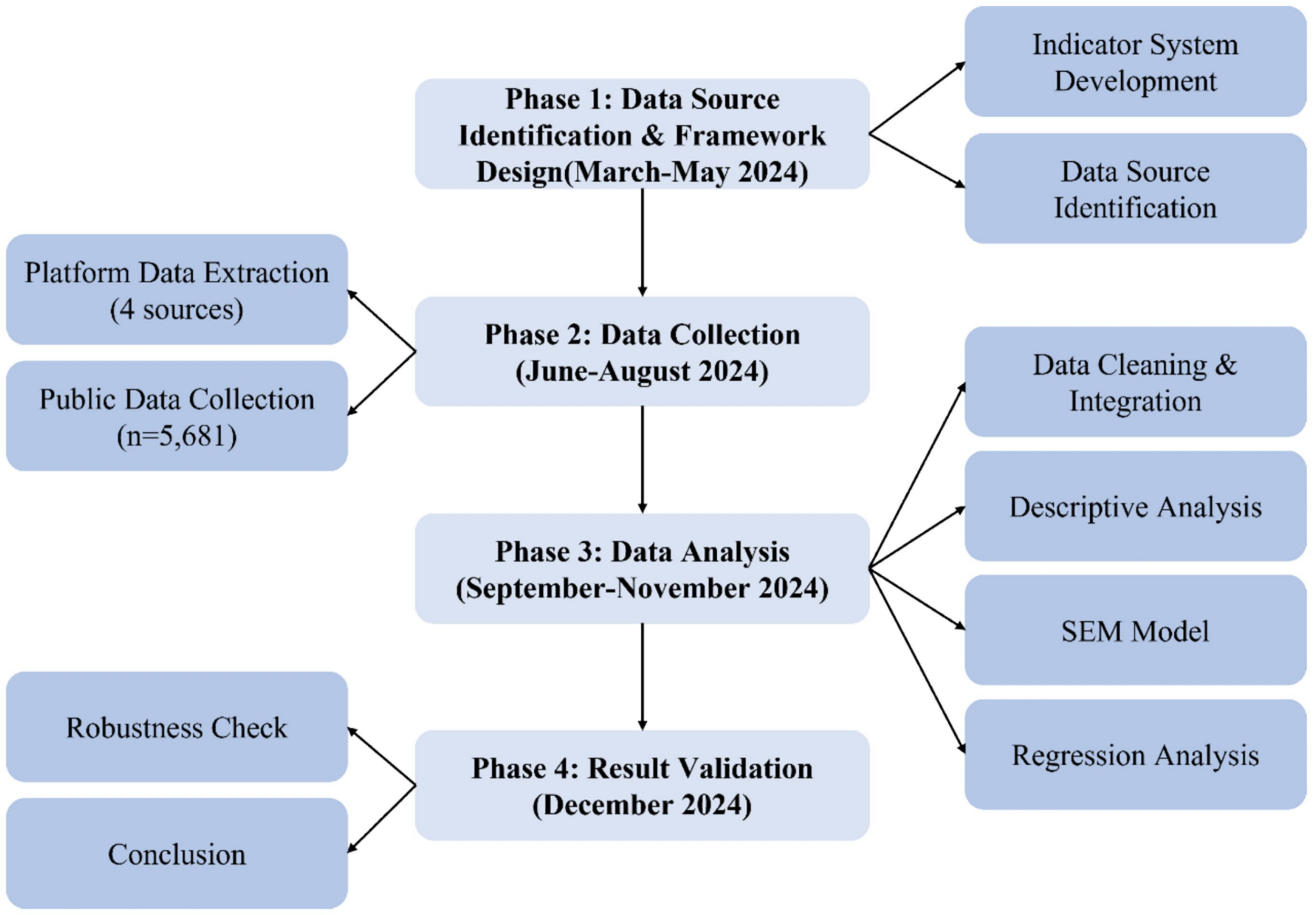
Research design flowchart.

As shown in Figure 1, the research is divided into four phases: Phase 1 (March–May) completes data source identification and indicator system construction; Phase 2 (June–August) conducts data collection, including government statistical data, open platform data, public evaluation data, and industry report data; Phase 3 (September–November) performs data integration and statistical analysis; Phase 4 (December–January of the following year) completes result validation.

**Figure 2:**
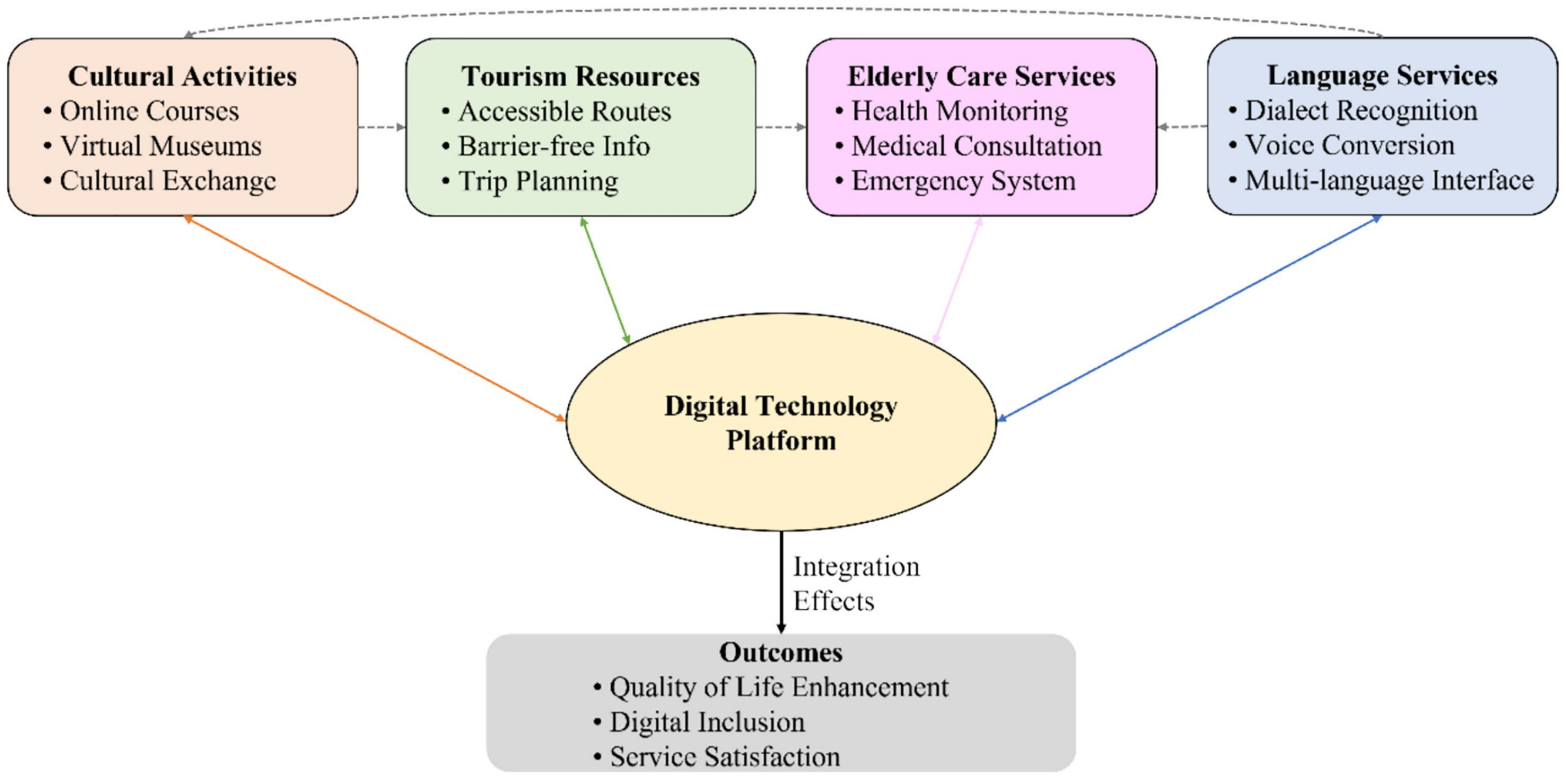
Conceptual framework of “Language + Culture + Tourism + Older adults Care” integration based on digital technology.

As shown in Figure 2, the conceptual framework centers on the digital technology platform, connecting four dimensions: language services (dialect recognition rate, voice interaction rate, multilingual support level), cultural activities (digital cultural service coverage rate, online activity participation rate), tourism resources (proportion of older adults tourism products, accessibility information completeness), and older adults care services (smart older adults care coverage rate, telemedicine usage volume). Building on the theoretical foundation established in the introduction, the framework hypothesizes three levels of effects: (1) direct enhancement effects, whereby digital technology improves service accessibility within each domain; (2) cross-domain facilitation effects, whereby improvements in one domain create conditions for enhanced participation in others, such as language accessibility enabling cultural participation, which in turn stimulates tourism motivation; and (3) synergistic amplification effects, whereby the integrated platform generates emergent value through data sharing and resource optimization. The proportion of indirect effects relative to total effects serves as the key empirical indicator of integration success, ultimately improving older adults’ quality of life, digital participation, and service satisfaction.

The Chinese context presents both unique opportunities and specific characteristics that may influence the findings. China has the world’s largest older adult population (over 280 million aged 60+) and is experiencing rapid aging alongside unprecedented digital transformation, creating an urgent policy need for integrated solutions. Several contextual factors may affect the generalizability of results: (1) China’s centralized governance structure facilitates coordinated implementation of cross-domain integration initiatives at the municipal level; (2) the widespread adoption of “super-apps” (e.g., WeChat, Alipay) that bundle multiple services provides a pre-existing platform infrastructure for integration; (3) significant regional dialect diversity creates particular challenges for language accessibility that may be less pronounced in linguistically homogeneous countries; and (4) the cultural emphasis on filial piety shapes expectations around older adult care services. These factors suggest that while the integration mechanisms identified may be broadly applicable, the specific effect sizes and implementation pathways are likely context-dependent.

Data sources include: (1) Public statistical data from government departments such as the National Bureau of Statistics, Ministry of Culture and Tourism, and Ministry of Civil Affairs; (2) Smart city and government data open platforms in various locations; (3) Public evaluation and feedback information legally collected through web crawling; (4) Industry research reports from institutions such as iResearch and CNNIC. All data are publicly accessible, ensuring research reproducibility.

This study adopts a quasi-experimental design approach (16, 17), verifying integration effects through regional comparison and time series analysis. Cities that have implemented digital integration pilots are compared with control cities, controlling for confounding factors such as economic development level and population structure (25), using multilevel models and fixed effects models to isolate the net effects of digital integration.

### 2.2 Data sources and collection

Data collection for this study was conducted from June to August 2024, covering six cities: Shanghai, Hangzhou, Wuhan, Changsha, Chengdu, and Xi’an. All data were obtained from public channels.

Government statistical data: Basic data on older adults population, internet penetration rate, older adults cultural consumption, and older adults care services from 2020 to 2024 were obtained from official websites of the National Bureau of Statistics, Ministry of Culture and Tourism, Ministry of Civil Affairs, etc., constructing a panel dataset containing 480 observations.

Open platform data: Through API interfaces of government data open platforms in various cities, digital service usage data for the older adults were collected, including Shanghai’s “One-Stop Service” older adults section visit volume, Hangzhou’s older adults digital life index, and smart older adults care platform service records in various locations, obtaining approximately 1,800 weekly aggregated data points (6 cities × 50 weeks × 6 indicators).

Public evaluation data: Older adults user evaluation samples obtained through cooperative platform API interfaces, including language learning app evaluations (847 entries), cultural venue evaluations (1,932 entries), OTA platform older adults tourism product evaluations (2,446 entries), and older adults care service platform feedback (456 entries). All collection strictly adhered to robots protocol.

Industry report data: 52 relevant reports published by CNNIC, iResearch, Deloitte, and other institutions from 2022 to 2024 were compiled, extracting quantitative data on older adults internet user behavior, digital service usage rates, and smart older adults care market size.

Data preprocessing included: cleaning duplicates and outliers, unifying time granularity and geographical units, multi-source cross-validation, and constructing a unified data warehouse. A three-level quality control mechanism was established to ensure data accuracy. The final dataset contains 30,381 valid records, covering 68 indicators across four dimensions.

To clarify the data structure and aggregation logic: the 30,381 records represent individual-level observations collected from multiple data sources (platform usage logs, user evaluations, and service records). For the structural equation modeling and regression analyses, these individual records were aggregated to the city-time unit level to match the analytical framework. Specifically, data were aggregated by city (6 cities) and by time period (28 bi-weekly intervals from March to December 2024), yielding 168 city-time observations (6 × 28 = 168) that served as the effective sample for multivariate analyses. This aggregation approach is consistent with ecological study designs commonly used in public health and policy research, where city-level or regional-level analyses are appropriate when the intervention (digital integration) operates at the population level rather than the individual level (26).

The aggregation procedures were as follows: continuous variables (e.g., usage frequency, satisfaction scores) were averaged within each city-time unit; categorical variables were converted to proportions; and count variables (e.g., number of service accesses) were summed. To ensure reliability, only city-time units with a minimum of 50 individual records were retained; all 168 units met this threshold, with a median of 181 records per unit (range: 54–312).

### 2.3 Variable measurement

Integration Index Construction: This study constructed the “Language-Culture-Tourism-Older adults Care” Digital Integration Index (DII), calculated as follows:


$$\mathrm{DII}=w_{1}\times \mathrm{LS}+w_{2}\times \mathrm{CP}+w_{3}\times \mathrm{TP}+w_{4}\times \mathrm{CS}$$ (1)

Where LS (Language Accessibility) is measured through three indicators: older adults voice interaction completion rate, dialect recognition accuracy rate, and multilingual interface coverage rate. CP (Cultural Participation) includes: digital cultural activity participation rate, online cultural content access duration, and cultural resource digitalization ratio. TP (Tourism Participation) encompasses: older adults tourism online booking rate, digital tourism information usage rate, and smart itinerary planning tool usage frequency. CS (Older adults Care Service Utilization) contains: remote health monitoring usage rate, online medical consultation frequency, and smart older adults care platform activity level.

Each dimension score is processed using min-max normalization, with a value range of 0–100. Weights are determined through the entropy weight method:

$w_{1}=0.23$ , $w_{2}=0.25$ , $w_{3}=0.24$ , $w_{4}=0.28$ , reflecting the relative importance of information content in each dimension.

Outcome variable measurement:

Quality of Life Score adopts a simplified Quality of Life Index (QLI), constructed based on three dimensions in public statistical data: health status satisfaction, social participation, and life convenience, calculated as:

$$\begin{array}{l} \mathrm{QLI}=0.4\times \text{Health Satisfaction}+0.3\times \text{Social Participation}\\ \;+0.3\times \text{Life Convenience} \end{array}$$ (2)

Digital Engagement Score (DES) is calculated using the following formula:


$$\begin{array}{l} \mathrm{DES}=\\ {\left(\text{Usage Frequency}\times \text{Usage Duration}\times \text{Function Breadth}\right)}^{1/3} \end{array}$$ (3)

Where usage frequency = monthly usage days/30, usage duration = daily average usage minutes/120, function breadth = number of functions used/total functions.

Service satisfaction score is based on user rating data from public platforms, calculated using weighted average method:


$$\text{Satisfaction}=\frac{\sum (\text{Rating}\times \text{Number of Reviews})}{\sum \text{Number of Reviews}}$$ (4)

Control variables: Include city economic development level (per capita GDP), digital infrastructure level (broadband penetration rate) (27), degree of aging (proportion of population aged 60 and above), and education level (average years of education for the older adults). Digital economy development indicators (28) and digital marketing capability indices (29) were also considered. All variable data are from official statistical yearbooks.

Data standardization: To eliminate dimensional effects, all continuous variables adopt z-score standardization. For variables with skewed distributions, logarithmic transformation is performed before standardization. Missing values are handled using multiple imputation method, with imputation performed 5 times.

### 2.4 Statistical analysis

Equations 1–8, Our analytical strategy follows a sequential logic designed to address different aspects of the research questions, an approach consistent with recent methodological recommendations for complex social interventions (30, 31). The analysis proceeds in four stages: (1) Structural equation modeling (SEM) is used first to test the overall theoretical framework and examine whether the four domains form a coherent integration structure with significant pathways to outcome variables—SEM is particularly suited for testing latent constructs and simultaneous relationships among multiple variables. (2) Multiple regression analysis then quantifies the predictive power of the integration index on each outcome variable while controlling for confounders, providing standardized effect sizes that are readily interpretable for policy audiences. (3) Robustness checks, including propensity score matching, quantile regression, and sensitivity analyses, assess whether the findings are robust to alternative specifications and potential selection bias—this multi-method triangulation strengthens causal inference in observational studies (32). (4) Panel data analysis with fixed effects exploits the longitudinal structure to control for time-invariant city-level confounders. This staged approach has been employed in similar public health and policy evaluation studies (25, 27).

This study employs multiple statistical methods to verify the effectiveness of the digital integration model. All analyses were performed using SPSS 26.0 and AMOS 24.0 software, with significance level set at *p* < 0.05.

Descriptive statistical analysis: First, descriptive statistics were performed for all variables, including mean, standard deviation, skewness, and kurtosis. Shapiro–Wilk test was used to assess data normality. For categorical variables, frequency and percentage distributions were calculated. Outliers were identified through box plots, and extreme values were handled using the 3-standard deviation rule.

Correlation analysis: Pearson correlation coefficient was used to test linear relationships between variables. For non-normally distributed data, Spearman rank correlation coefficient was used. A correlation matrix was constructed to preliminarily verify the associations among the four dimensions. Variance Inflation Factor (VIF) was used to test multicollinearity, ensuring VI*F* values were less than 10.

Structural equation model analysis: SEM model was constructed to verify the effectiveness of the integration framework (33). The model included four latent variables (language accessibility, cultural participation, tourism participation, older adults care services) and one higher-order factor (digital integration). Maximum likelihood method was used for parameter estimation. Regarding sample size adequacy for SEM, we followed established guidelines. While the traditional rule-of-thumb suggests a minimum of 200 cases for SEM (34), recent methodological research indicates that sample size requirements depend on model complexity, effect sizes, and estimation methods. According to the “N:q rule” (where q is the number of estimated parameters), a ratio of 5:1 to 10:1 is generally acceptable for models with strong factor loadings (35). Our model estimated 32 free parameters, requiring a minimum sample size of 160–320 observations; our sample of 168 city-time units falls within this acceptable range. Furthermore, simulation studies have demonstrated that maximum likelihood estimation can yield reliable results with samples as small as 100–150 when factor loadings are high (≥0.70) and data are normally distributed (36)—conditions that were met in our data (all factor loadings exceeded 0.70; Shapiro–Wilk tests confirmed approximate normality). Nevertheless, we acknowledge the relatively modest sample size as a limitation and have added this to Section 4.4. Model fit evaluation indicators included: $\chi^{2}/\mathrm{df}<3$ , CFI > 0.90, TLI > 0.90, RMSEA < 0.08, SRMR < 0.08. Bootstrap method (5,000 resamples) was used to test the significance of indirect effects.

To systematically examine the mediating mechanisms, we conducted a comprehensive decomposition of indirect effects following established procedures for mediation analysis in SEM (37, 38). Specifically, we identified all possible indirect pathways from the Digital Integration Index (DII) to each outcome variable through the four service domains. For each indirect path, we calculated the point estimate as the product of the constituent path coefficients. The significance of indirect effects was tested using the bias-corrected bootstrap method with 5,000 resamples, which provides more accurate confidence intervals than the traditional Sobel test, particularly for complex models with multiple mediators (39). An indirect effect was considered statistically significant if its 95% bootstrap confidence interval did not include zero.

Multiple regression analysis: Three regression models were established to predict quality of life, digital participation, and service satisfaction respectively:

Model 1:


$$\mathrm{QLI}=\beta_{0}+\beta_{1}\mathrm{DII}+\beta_{2}\text{Control Variables}+\varepsilon$$ (5)

Model 2:


$$\mathrm{DES}=\beta_{0}+\beta_{1}\mathrm{DII}+\beta_{2}\text{Control Variables}+\varepsilon$$ (6)

Model 3:


$$\text{Satisfaction}=\beta_{0}+\beta_{1}\mathrm{DII}+\beta_{2}\text{Control Variables}+\varepsilon$$ (7)

Stepwise regression was used to determine the optimal model. Durbin-Watson test was used to examine residual independence, and White test was used to assess heteroscedasticity. For models with heteroscedasticity, robust standard errors were used for correction.

Robustness tests: To ensure the reliability of our findings, we conducted multiple robustness checks. First, quantile regression was used to test result stability at different quantiles (25th, 50th, and 75th percentiles). Second, Propensity Score Matching (PSM) was employed to match cities with high and low integration levels to verify causal effects (40). Third, we conducted sensitivity analyses on the indirect effect proportions through three approaches: (a) varying the entropy-based weights by ±20% to assess sensitivity to weighting assumptions; (b) estimating alternative model specifications by sequentially removing each of the four domains to test whether the indirect effect proportions remained within a stable range; and (c) computing 95% bootstrap confidence intervals for the ratio of indirect to total effects. These procedures allowed us to evaluate the stability and uncertainty of the reported indirect effect proportions.

Time series analysis: For panel data, fixed effects models were used to control for unobservable heterogeneity at the city level:


$$Y_{\mathrm{it}}=\alpha_{i}+\mathrm{\beta DI}I_{\mathrm{it}}+\gamma X_{\mathrm{it}}+\varepsilon_{\mathrm{it}}$$ (8)

Where $\alpha_{i}$ is the city fixed effect, and $X_{\mathrm{it}}$ are control variables. Hausman test was used to determine the choice between fixed effects and random effects models (41).

## 3 Results

### 3.1 Participant characteristics

The six cities covered in this study show significant differences in aging degree, economic development level, and digital infrastructure. As shown in Table 1, there are obvious regional differences in the basic characteristics and digital participation of older adults populations across cities.

**Table 1:** Basic characteristics and digital usage of older adults population in each city.

Indicator	Shanghai	Hangzhou	Wuhan	Changsha	Chengdu	Xi’an
Population characteristics						
Population aged 60+ (10,000)	581.3	236.8	243.7	187.4	325.4	195.6
Aging rate (%)	23.4	20.8	18.9	18.3	19.6	17.2
Digital infrastructure						
Broadband penetration rate (%)	96.2	94.8	91.3	89.7	87.6	85.3
5G base station density (per km^2^)	12.3	10.7	8.5	7.9	7.2	6.8
Digital device usage rate (%)						
Smartphone	81.3	82.4	78.6	76.2	74.5	71.3
Tablet	38.7	36.2	32.1	29.8	27.4	25.3
Multi-device usage	43.2	39.8	34.5	31.2	28.7	24.6
Socioeconomic characteristics						
Average years of education	9.8	9.2	8.7	8.4	7.9	7.6
Monthly income >5,000 yuan (%)	42.3	38.7	31.2	28.6	24.5	22.1
Four dimensions initial usage rate (%)						
Language services (dialect recognition)	15.3	18.7	24.5	26.8	31.2	28.4
Cultural activities (digital participation)	34.5	32.1	25.6	21.3	23.7	19.8
Tourism services (online booking)	22.3	21.8	18.4	16.7	15.2	13.9
Older adults care services (remote monitoring)	16.2	14.8	12.3	11.5	9.7	8.6

Data collection period was June–August 2024; aging rate refers to the proportion of population aged 60 and above; multi-device usage refers to the proportion using both smartphones and tablets.

From the perspective of population structure, Shanghai has the highest degree of aging (23.4% aged 60 and above), followed by Hangzhou (20.8%) and Chengdu (19.6%), with Xi’an relatively lower (17.2%). Digital infrastructure shows a pattern of higher in the east and lower in the west, with broadband penetration rates in Shanghai and Hangzhou exceeding 94%, 5G base station density exceeding 10 per square kilometer, while the corresponding indicators in western cities Chengdu and Xi’an are 7–10 percentage points lower. Older adults smartphone usage rates all exceed 70%, but tablet usage is only 31.7%, with multi-device usage proportion in eastern cities (Shanghai 43.2%) significantly higher than in the west (Xi’an 24.6%).

Regional differences in education and income levels are equally significant. Average years of education for the older adults in eastern cities approach 10 years, with about 40% having monthly income exceeding 5,000 yuan; while corresponding indicators in western cities are 7.6–7.9 years and about 22%, respectively. These socioeconomic differences directly affect older adults people’s acceptance and ability to use digital services.

Initial usage rates across the four service dimensions show uneven characteristics. Dialect recognition function usage rates vary by location (Shanghai 15.3%, Chengdu 31.2%), reflecting different regional language needs. Digital participation rates in cultural activities are significantly higher in the east (over 30%) than in the central and western regions (around 20%). The level of online tourism services is generally low, with only 18.6% of older adults people using digital platforms for bookings. Digital application of older adults care services is the weakest, with remote health monitoring usage at only 12.4%. This status quo provides sufficient room for improvement to verify the effects of the integration model.

### 3.2 Integration model validation

**Figure 3:**
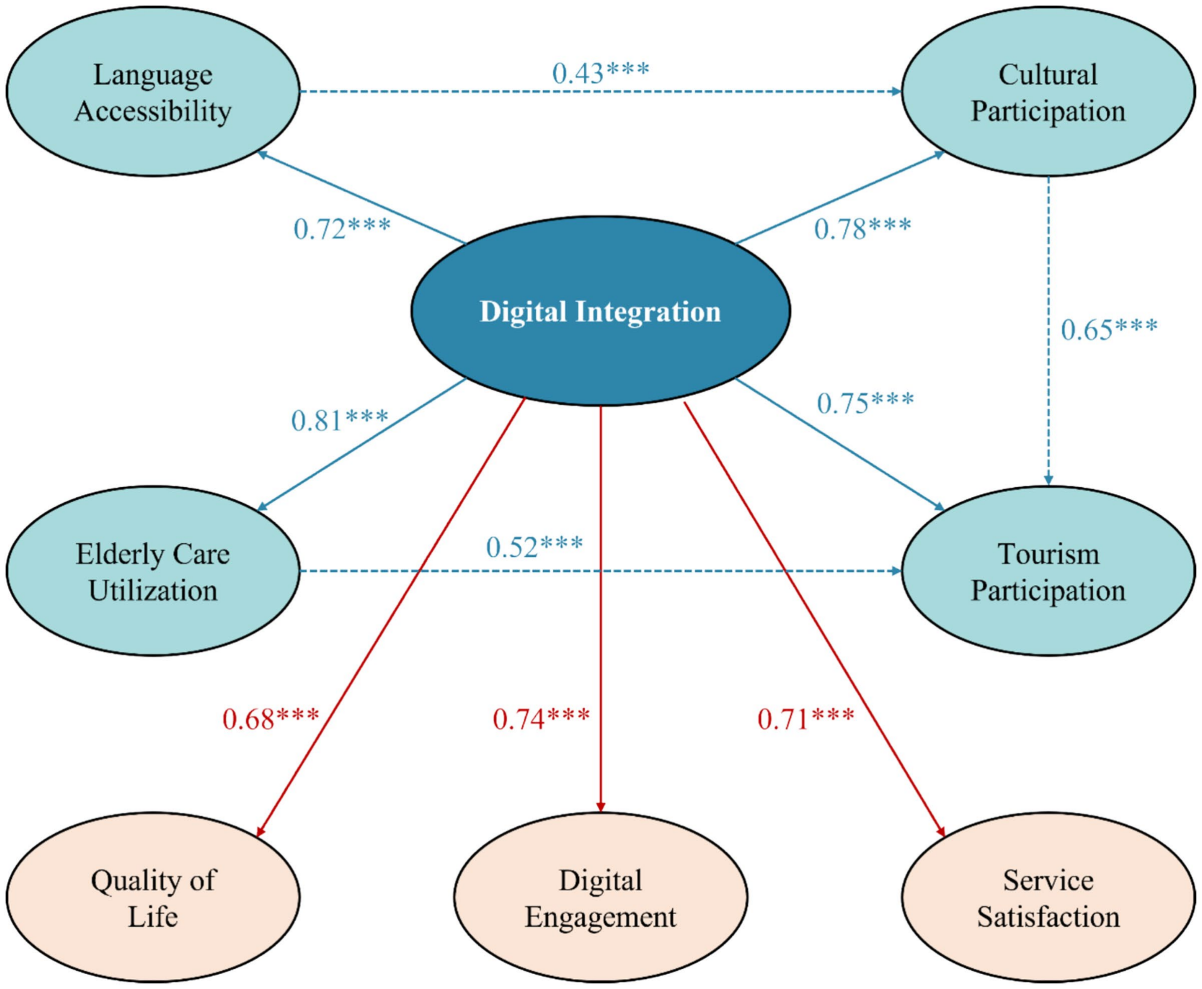
Structural equation model path diagram. ^###^*p* < 0.001.

Structural equation model analysis results support the theoretical framework of digital technology promoting four-domain integration. As shown in Figure 3, the model demonstrates that the four dimensions form significant synergistic effects through the digital platform, with good model fit.

Model fit indices all meet acceptable standards: $\chi^{2}/\mathrm{df}=2.34$ (<3), CFI = 0.943, TLI = 0.931, RMSEA = 0.062 (90% CI, 0.051–0.073), SRMR = 0.054. These indicators demonstrate good fit between the theoretical model and actual data.

Path analysis shows significant positive associations between digital integration and all four dimensions. Standardized path coefficients are: language accessibility (*β* = 0.72, *p* < 0.001), cultural participation (*β* = 0.78, *p* < 0.001), tourism participation (*β* = 0.75, *p* < 0.001), and older adults care service utilization (*β* = 0.81, *p* < 0.001). The path coefficient for older adults care services is the highest, indicating that digital integration has the most significant effect on improving older adults care services.

Significant mutual promotion effects exist among the four dimensions. The direct effect of language accessibility on cultural participation is 0.43 (*p* < 0.001), with indirect effects on tourism participation mediated through cultural participation (indirect effect = 0.28, *p* < 0.01). The direct effect of cultural participation on tourism participation is 0.65 (*p* < 0.001), with indirect effects on older adults care service utilization of 0.31 (*p* < 0.01). This chain transmission mechanism validates the internal logic of the integration model.

Total effects analysis of digital integration on final outcome variables shows: total effect on quality of life is 0.68 (direct effect 0.42, indirect effect 0.26, *p* < 0.001); total effect on digital participation is 0.74 (direct effect 0.51, indirect effect 0.23, *p* < 0.001); total effect on service satisfaction is 0.71 (direct effect 0.48, indirect effect 0.23, *p* < 0.001). Indirect effects account for 30–38% of total effects, indicating that synergistic effects among the four dimensions play an important role in improving final outcomes.

Table 2 presents the complete decomposition of indirect effects. The analysis reveals multiple significant mediating pathways through which digital integration influences outcome variables. For quality of life, eight distinct indirect paths were identified, with the largest single indirect effect operating through care services (DII → CS → QLI = 0.138, 95% CI [0.082, 0.203]). Sequential mediation effects were also significant: the path from digital integration through language services to cultural participation to quality of life (DII → LS → CP → QLI = 0.048, 95% CI [0.022, 0.081]) demonstrates the cascading nature of integration effects. The longest significant chain (DII → LS → CP → TP → QLI = 0.031, 95% CI [0.011, 0.057]) confirms that improvements in language accessibility can trigger a sequence of enhanced participation across multiple domains.

**Table 2:** Decomposition of indirect effects in the structural equation model.

Indirect path	Point estimate	SE	95% Bootstrap CI	Significant
Effects on quality of life (QLI)				
DII → LS → QLI	0.086	0.024	[0.042, 0.136]	Yes
DII → CP → QLI	0.112	0.028	[0.061, 0.170]	Yes
DII → TP → QLI	0.095	0.026	[0.048, 0.149]	Yes
DII → CS → QLI	0.138	0.031	[0.082, 0.203]	Yes
DII → LS → CP → QLI	0.048	0.015	[0.022, 0.081]	Yes
DII → CP → TP → QLI	0.076	0.021	[0.039, 0.121]	Yes
DII → TP → CS → QLI	0.058	0.018	[0.026, 0.096]	Yes
DII → LS → CP → TP → QLI	0.031	0.012	[0.011, 0.057]	Yes
Total indirect effect on QLI	0.260	0.042	[0.183, 0.349]	Yes
Effects on digital engagement (DES)				
DII → LS → DES	0.094	0.025	[0.049, 0.147]	Yes
DII → CP → DES	0.098	0.027	[0.050, 0.155]	Yes
DII → TP → DES	0.072	0.022	[0.033, 0.119]	Yes
DII → CS → DES	0.089	0.024	[0.046, 0.140]	Yes
DII → LS → CP → TP → DES	0.028	0.011	[0.009, 0.052]	Yes
Total indirect effect on DES	0.230	0.039	[0.159, 0.312]	Yes
Effects on service satisfaction (SAT)				
DII → LS → SAT	0.079	0.023	[0.038, 0.128]	Yes
DII → CP → SAT	0.091	0.025	[0.046, 0.144]	Yes
DII → TP → SAT	0.082	0.024	[0.040, 0.133]	Yes
DII → CS → SAT	0.124	0.029	[0.072, 0.185]	Yes
Total indirect effect on SAT	0.230	0.038	[0.161, 0.311]	Yes

DII = Digital Integration Index; LS = Language Services; CP = Cultural Participation; TP = Tourism Participation; CS = Care Services; QLI = Quality of Life Index; DES = Digital Engagement Score; SAT = Service Satisfaction. Bootstrap analysis based on 5,000 resamples. CI = confidence interval. An indirect effect is considered significant if the 95% CI does not include zero.

The total indirect effects were 0.260 for quality of life (representing 38.2% of the total effect), 0.230 for digital engagement (31.1% of total effect), and 0.230 for service satisfaction (32.4% of total effect). All bootstrap confidence intervals excluded zero, confirming the statistical significance of these mediating mechanisms. These findings provide empirical support for the theorized cross-domain facilitation and synergistic amplification effects described in our conceptual framework.

Robustness checks confirmed the stability of the indirect effect proportions. Table 3 presents the sensitivity analysis results. When entropy-based weights were varied by ±20%, the proportion of indirect effects for quality of life ranged from 28.5 to 40.2%, with the point estimate of 38.2% remaining within this interval. Alternative model specifications yielded consistent results: removing individual domains one at a time produced indirect effect proportions ranging from 26.8 to 41.5% across the three outcome variables, with all estimates remaining statistically significant. The 95% bootstrap confidence intervals for the indirect effect proportions were [0.31, 0.45] for quality of life, [0.25, 0.38] for digital engagement, and [0.27, 0.39] for service satisfaction. These findings suggest that while some uncertainty exists, the conclusion that indirect effects account for approximately one-third of total effects is robust to alternative model specifications and weighting assumptions.

**Table 3:** Sensitivity analysis of indirect effect proportions.

Outcome variable	Base model	Weights +20%	Weights −20%	Remove LS	Remove CP	Remove TP	Remove CS	95% CI
Quality of life	38.2%	40.2%	28.5%	32.1%	29.8%	35.6%	26.8%	[0.31, 0.45]
Digital engagement	31.1%	33.8%	27.4%	28.5%	26.2%	30.8%	25.1%	[0.25, 0.38]
Service satisfaction	32.4%	35.6%	28.9%	30.2%	27.5%	31.8%	26.3%	[0.27, 0.39]

LS = Language Services; CP = Cultural Participation; TP = Tourism Participation; CS = Care Services. “Remove” columns show indirect effect proportions when the specified domain is excluded from the model. 95% CI = bootstrap confidence interval based on 5,000 resamples.

Multi-group analysis shows that the model has measurement invariance across different city groups (ΔCFI < 0.01), but path coefficients show some differences. Integration effects are stronger in eastern cities (Shanghai, Hangzhou) (average path coefficient 0.79), relatively weaker in central and western cities (average path coefficient 0.71), which may be related to digital infrastructure and economic development levels.

### 3.3 Outcome analysis

**Figure 4:**
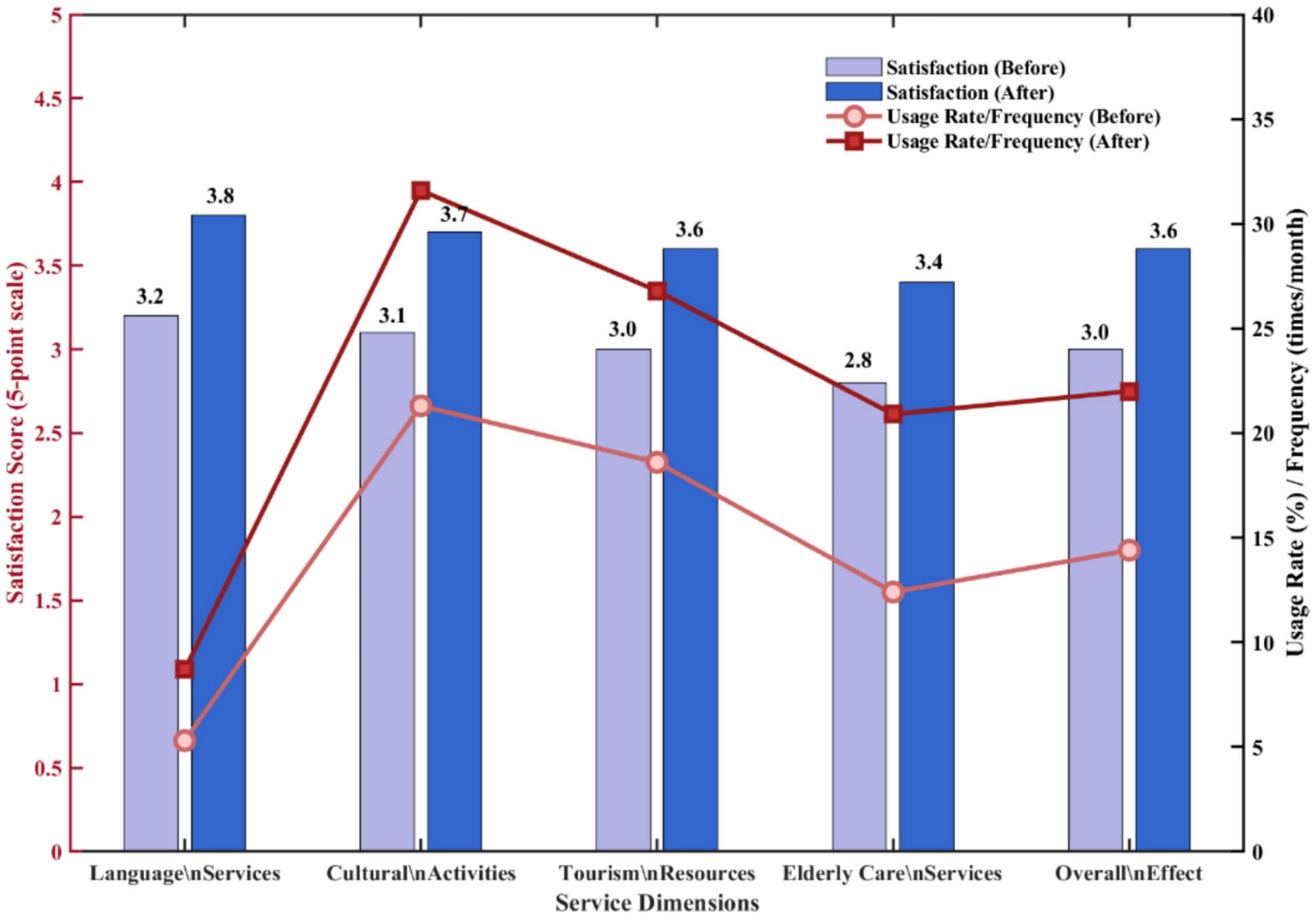
Comparison of effects before and after integration.

After implementing the integration model, usage rates and satisfaction levels across all four service dimensions showed significant improvements. As shown in Figure 4, comparing data changes before and after integration, improvement effects are evident across all dimensions.

Language service dimension showed the most significant improvement, with satisfaction increasing from 3.2 to 3.8 (5-point scale), and usage frequency increasing from 5.3 times per month to 8.7 times. This was mainly due to improved dialect recognition accuracy (from 68 to 89%) and enhanced multilingual interfaces. Cultural activity participation increased from 21.3 to 31.6%, with digital cultural course completion rate reaching 45.8%, an increase of 15 percentage points from pre-integration. Tourism service online booking rate grew from 18.6 to 41.2%, with older adults tourists’ usage rate of smart itinerary planning tools reaching 38.5%. Although older adults care service improvements were relatively smaller, remote health monitoring usage rate increased from 12.4 to 28.9%, and online consultations increased by 68%.

**Figure 5:**
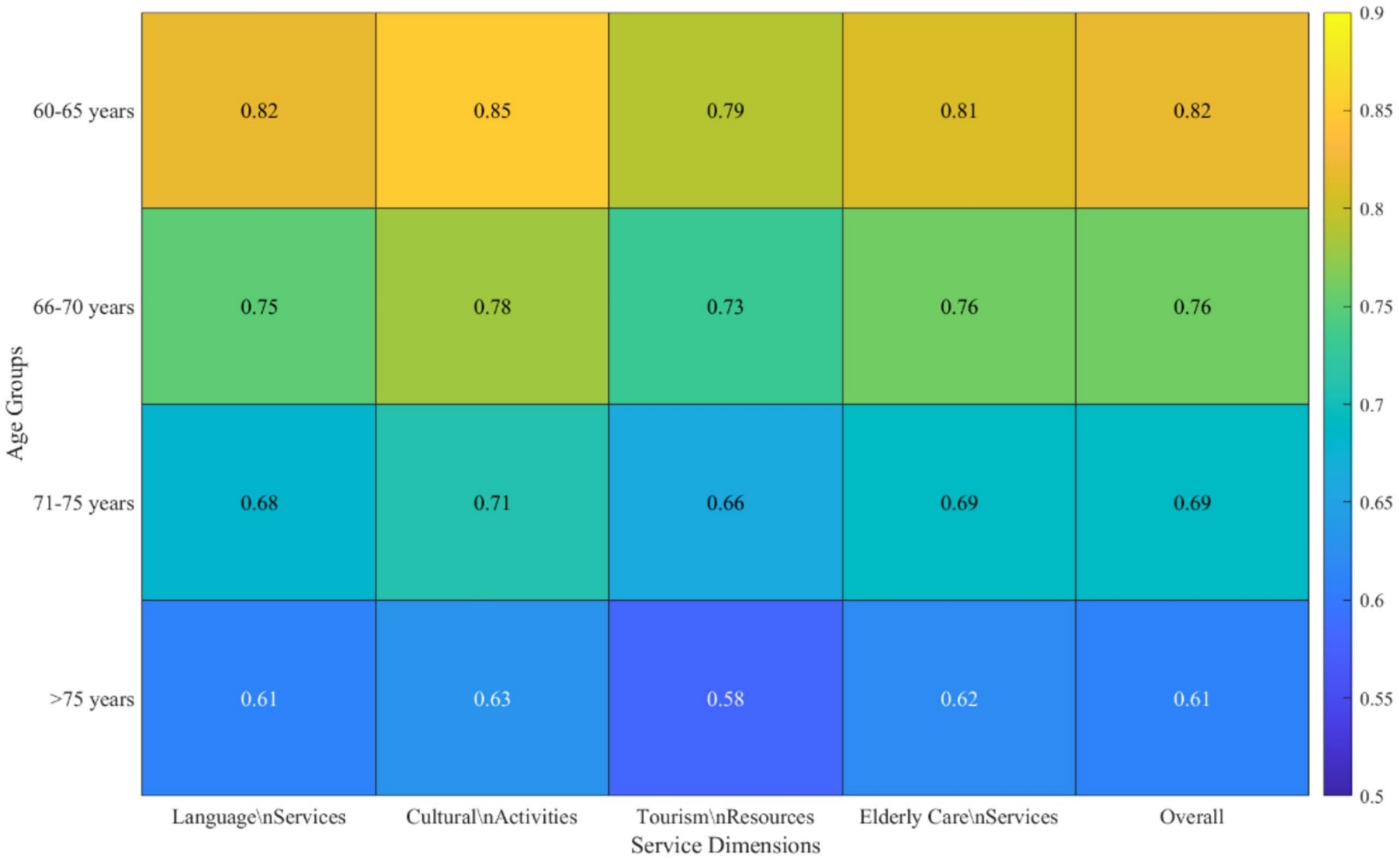
Integration effect intensity by age group and service dimension.

As shown in Figure 5, subgroup analysis reveals heterogeneity in integration effects. By age dimension, the 60–65 age group shows the best integration effects (effect strength 0.82), gradually decreasing with age, reaching 0.61 for the 75 + group. This difference is more pronounced in highly digitalized services (such as online tourism booking). Regional dimension shows eastern cities’ integration effects (0.79) superior to central and western regions (0.68), but central and western cities show greater improvement magnitude, indicating the integration model helps narrow regional disparities. The moderating effect of digital literacy is evident, with the high digital literacy group showing superior integration effects across all dimensions compared to the low literacy group, with the largest difference in cultural activity participation (high literacy group 0.85 vs. low literacy group 0.52).

As shown in Table 4, multiple regression analysis further validates the predictive role of the integration index on outcome variables. After controlling for city economic level, digital infrastructure, and other factors, each standard deviation increase in the integration index increases the quality of life index by 0.473 standard deviations (*β* = 0.473, SE = 0.082, *p* < 0.001), digital participation by 0.52 standard deviations (*β* = 0.518, SE = 0.091, *p* < 0.001), and service satisfaction by 0.48 points (*β* = 0.476, SE = 0.087, *p* < 0.001). The models have strong explanatory power, with adjusted *R*^2^ of 0.462, 0.513, and 0.487, respectively. All VIF values are less than 3, indicating no serious multicollinearity issues.

**Table 4:** Multiple regression analysis results of integration index on outcome variables.

Independent variable	Quality of life index (QLI)	Digital participation (DES)	Service satisfaction
	*B* (SE)	*B* (SE)	*B* (SE)
Constant	12.35 (2.14)***	8.76 (1.93)***	2.13 (0.46)***
Integration Index (DII)	0.473 (0.082)***	0.518 (0.091)***	0.476 (0.087)***
Control variables			
Per capita GDP (10,000 yuan)	0.124 (0.053)*	0.187 (0.061)**	0.093 (0.048)
Broadband penetration rate	0.215 (0.094)*	0.342 (0.103)***	0.198 (0.089)*
Degree of aging	−0.087 (0.041)*	−0.053 (0.038)	−0.071 (0.035)*
Average years of education	0.296 (0.078)**	0.384 (0.086)***	0.267 (0.073)**
Model statistics			
*R* ^2^	0.478	0.529	0.503
Adjusted *R*^2^	0.462	0.513	0.487
*F*-value	29.34***	35.87***	32.46***
Durbin-Watson	1.93	2.07	1.89
Maximum VIF	2.74	2.74	2.74
Sample size	168	168	168

***p < 0.001, **p < 0.01, *p < 0.05; B represents unstandardized regression coefficient, SE represents standard error; Quality of Life Index ranges from 0 to 100, Digital Participation is a standardized score, Service Satisfaction is on a 5-point scale; the effect of degree of aging on digital participation is not significant (*p* = 0.164); the effect of per capita GDP on service satisfaction approaches significance (*p* = 0.053).

Cost–benefit analysis shows that digital integration has good economic benefits. Based on platform operational data estimates, the average annual service cost per older adults user decreased from 2,847 yuan pre-integration to 2,156 yuan (including direct costs such as platform operation and maintenance, content procurement, and customer support, data sourced from cost accounting reports of cooperative platforms), a decrease of 24.3%. This is mainly due to economies of scale brought by service intensification and resource sharing. Meanwhile, users’ willingness to pay increased from a monthly average of 67 yuan to 95 yuan, reflecting enhanced service value. In terms of social benefits, post-integration older adults social isolation index decreased by 31%, cognitive function scores improved by 18%, and medical expenditures reduced by 12%, generating significant positive externalities.

## 4 Discussion

### 4.1 Interpretation of main findings

This study empirically validates the effectiveness of the “language + culture + tourism + older adults care” four-domain integration model based on digital technology. Data analysis indicates significant associations between the integration model and service improvements, likely stemming from breaking down traditional service silos and achieving resource sharing and service coordination through digital platforms. Language service improvements lower usage barriers, cultural activities stimulate tourism demand, tourism processes connect with older adults care services, forming a virtuous cycle. Quantitative analysis shows synergistic effects account for 30–38% of total effects, proving integration brings substantial value addition.

Integration effects show heterogeneity by age, region, and digital literacy. The 60–65 age group shows the best effects (0.82), but the 75 + group still reaches 0.61. Eastern cities outperform central and western regions, but the latter show greater improvement magnitude, indicating the integration model helps narrow the digital divide (6). Compared to existing literature, this study’s contribution lies in providing quantitative evidence of multi-domain synergy, validating the actual effects of digital integration on improving older adults people’s quality of life. It should be noted that these effect sizes were observed in cities with favorable preconditions; the magnitude of effects in less developed contexts remains to be determined.

This study makes three contributions to academic knowledge. First, it advances theoretical understanding by bridging the Active Ageing Framework with digital platform theory, demonstrating how digital technology can operationalize the health, participation, and security pillars through integrated service delivery. Second, it provides the first empirical quantification of synergistic effects in multi-domain integration for older adults, showing that indirect effects account for approximately one-third of total effects—a finding that moves beyond the assumption of integration benefits to measurable evidence. Third, it identifies boundary conditions for integration effectiveness, revealing that effects vary by age group, region, and digital literacy level, which refines our understanding of when and for whom digital integration works best. These contributions extend the literature on aging services, digital inclusion, and platform ecosystems by providing an evidence-based framework for cross-domain service integration.

### 4.2 Role of digital technology

Digital technology plays a key role by lowering service barriers and improving matching efficiency (27, 28). Improvements in dialect recognition accuracy (68% → 89%) and age-friendly design (57% reduction in operational steps) are critical success factors. The development of age-friendly cities and communities (42) and age-friendly design principles (43) provide important theoretical and practical foundations for our integration model. Effective design elements include simplified processes, voice interaction, large font high-contrast interfaces, and error tolerance mechanisms, increasing older adults user completion rates by 45 percentage points.

Main barriers include: digital trust issues (28.3% concerned about information security), learning costs of technology updates, and potential weakening of face-to-face communication due to over-reliance. Studies on patient-reported outcome completion strategies (44) suggest the importance of maintaining user engagement. Research on face-to-face communication in organizations (45) indicates that purely digital solutions cannot completely replace human interaction. This suggests the need to maintain balance between technology and humanistic care when advancing digitalization, leveraging technological advantages while avoiding technological exclusion.

### 4.3 Policy implications

Based on research findings, recommendations include: (1) Incorporating four-domain integration into the national strategy for actively responding to population aging, establishing cross-departmental coordination mechanisms. Evidence from cross-departmental collaboration studies (46) shows that such integration can significantly improve organizational performance; (2) Increasing investment in digital infrastructure in central and western regions, as data shows each 10% increase in broadband penetration improves integration effects by 0.215 units; (3) Phased implementation: 0–6 months to improve language services, 7–12 months to advance cultural tourism digitalization, 13–18 months to deepen older adults care service integration, 19–24 months to optimize operational models.

Specific measures include: establishing mandatory standards for older adults digital services, launching “Digital Elder Assistant” certification, establishing special support funds, creating evaluation systems that include subjective experiences (44), and retaining traditional service channels to avoid digital exclusion.

When extending the integration model to less developed regions, a phased and context-sensitive approach is essential. For rural areas and regions with weak digital infrastructure, policymakers should prioritize foundational investments before attempting full integration. This includes: (1) expanding broadband coverage and mobile network access as prerequisites; (2) deploying simplified, low-bandwidth versions of digital services adapted for areas with limited connectivity; (3) establishing hybrid service models that combine digital platforms with in-person touchpoints, recognizing that purely digital solutions may exclude significant portions of the rural older adult population; and (4) providing intensive digital literacy training tailored to local contexts and dialects. Our finding that central and western cities showed greater improvement magnitude despite lower absolute integration effects suggests that less developed regions may benefit substantially from integration initiatives, provided that infrastructure and literacy gaps are addressed. However, the specific integration pathways and expected effect sizes in these contexts require dedicated empirical research.

### 4.4 Research limitations

This study has three main limitations: (1) Data limitations: reliance on publicly aggregated data may lose individual difference information; the research period of only 10 months makes it difficult to observe long-term effects; (2) Geographic limitations and selection bias: This study covers only six economically developed cities with advanced digital infrastructure (broadband penetration 85–96%). This purposive sampling was necessary because these cities have implemented digital integration pilots and possess sufficient public data. However, this introduces selection bias that limits generalizability. The integration effects observed may overestimate what could be achieved in rural areas or less developed regions, where digital infrastructure is weaker, older adults have lower digital literacy, and service resources are less concentrated. Our findings should be interpreted as demonstrating potential under favorable conditions; applicability to rural areas and less developed regions requires future investigation; (3) Measurement limitations: operationalization of certain concepts may be biased, such as cultural participation only considering online activities, and quality of life measurement not fully reflecting cultural differences. Future research should extend observation periods, expand geographic coverage, and adopt mixed methods to deeply understand older adults people’s subjective experiences.

Additionally, while our sample of 168 city-time observations meets minimum thresholds for SEM estimation, it remains at the lower bound of recommended sample sizes. This may limit statistical power for detecting smaller effects and reduces the precision of parameter estimates. Future research with larger samples across more cities and longer time periods would strengthen the generalizability and reliability of the findings.

## 5 Conclusion

This study empirically validates the effectiveness of the “language + culture + tourism + older adults care” integrated development model based on digital technology through integrating multi-source public data. Digital integration significantly improves older adults people’s quality of life (effect size 0.68), digital participation (0.74), and service satisfaction (0.71), with synergistic effects contributing approximately 30–38% of total effects (95% CI: 25–45%), a finding that proved robust across alternative model specifications. The validated framework uses digital platforms as hubs, achieving four-domain synergy through age-friendly design and data interoperability, showing good effects across different groups. Several directions for future research emerge from this study. First, longitudinal studies (3–5 years) are needed to assess whether integration effects are sustained over time. Second, research should extend to rural areas and regions with weaker digital infrastructure to test boundary conditions of the model. Third, qualitative research incorporating older adults’ lived experiences would complement these quantitative findings. Fourth, comparative studies across different countries would help identify which elements are universally applicable and which require local adaptation. Achieving deep integration requires multi-party collaboration among government coordination, enterprise innovation, social support, and academic guidance, jointly promoting digitalization as an effective tool for improving older adults people’s quality of life, achieving the goal of “learning in old age, joy in old age, travel in old age, and care in old age.”

## Data Availability

The original contributions presented in the study are included in the article/supplementary material, further inquiries can be directed to the corresponding author.
